# Protective Effects of Aqueous Extract of *Mentha suaveolens* against Oxidative Stress-Induced Damages in Human Keratinocyte HaCaT Cells

**DOI:** 10.1155/2019/5045491

**Published:** 2019-09-22

**Authors:** HansongI Lee, Miji Yeom, Seoungwoo Shin, Kyungeun Jeon, Deokhoon Park, Eunsun Jung

**Affiliations:** Biospectrum Life Science Institute, A-1805, U-Tower, 767, Sinsu-ro, Suji-gu, 16827 Yongin-si, Gyeonggi-do, Republic of Korea

## Abstract

*Mentha suaveolens* is an aromatic herb that has a wide range of biological activities, including antimicrobial, antifungal, anti-inflammatory, and hepatoprotective properties. Although there are a few reports on the antioxidant property of *M. suaveolens*, its cytoprotective activity against oxidative stress has not been reported yet. The objective of this study was to determine the protective activity of *M. suaveolens* aqueous extract (MSAE) against hydrogen peroxide- (H_2_O_2_-) induced oxidative stress and apoptosis in human keratinocyte HaCaT cells. MSAE pretreatment decreased H_2_O_2_-induced cytotoxicity and suppressed H_2_O_2_-induced intracellular ROS generation. Furthermore, MSAE suppressed expression levels of H_2_O_2_-induced apoptotic genes such as cleaved caspase-3, caspase-9, and cleaved poly (ADP-ribose) polymerase (PARP). Pretreatment with MSAE induced expression of phase II enzyme such as HO-1 through translocation of NF-E2-related factor (Nrf2) upon H_2_O_2_ exposure. These results revealed that the cytoprotective effect of MSAE against oxidative stress-induced cell death was associated with activation of Nrf2-mediated phase II enzyme expression.

## 1. Introduction

Skin, the largest organ in human body, has direct contact with physical and chemical environmental factors. When skin cells are exposed to environmental stimuli, reactive oxygen species (ROS) are generated at cellular level [1]. The production of ROS such as superoxide anion, hydroxyl radical, and hydrogen peroxide is the most important mediator of oxidative stress. It triggers apoptosis [2, 3]. In skin cells, Kelch-like ECH-associated protein 1 (Keap1)-nuclear factor erythroid 2-related factor 2 (Nrf2) pathway is a key defense mechanism against oxidative stress [4]. Under normal physiological conditions, Nrf2 exists in the cytoplasm in complex with Keap1. However, under conditions of oxidative stress, Nrf2 will dissociate with Keap1, translocate into the nucleus, and bind to antioxidant response elements (ARE) present in the promoter region of antioxidant and detoxification genes including glutamate-cysteine ligase (GCL), quinone oxidoreductase-1 (NQO1), and heme oxygenase-1 (HO-1) [5, 6]. HO-1 produces carbon monoxide (CO), biliverdin, and iron by catalysis of heme. These products have protective roles against apoptosis [7, 8].

*Mentha suaveolens*, an aromatic herb commonly known as apple mint, belongs to family Lamiaceae. It is used as an herbal tea or food additive because of its unique aroma and flavor. *M. suaveolens* has a wide range of effects such as stimulating, stomachic, choleretic, analgesic, and antidiarrheal effects. Because of these benefits, it has been used as folk medicine [9, 10]. Furthermore, it has been reported that it possesses various biological properties such as antioxidant, antimicrobial, antifungal, anti-inflammatory, and anticancer [11–13]. In a previous study, we have observed that *M. suaveolens* extract exerted the antithermal skin-aging effect in human dermal fibroblasts [14]. However, the cytoprotective activity of *M. suaveolens* against oxidative stress-induced cell damage in keratinocytes has not been reported yet.

Therefore, the objective of this study was to investigate whether *M. suaveolens* aqueous extract (MSAE) could ameliorate hydrogen peroxide- (H_2_O_2_-) induced cell damages in keratinocytes. To further assess the cytoprotective effect of MSAE, we hypothesized that cytoprotective effect of *M. suaveolens* aqueous extract (MSAE) was associated with Nrf2 and the expression of antioxidant and detoxification enzymes. Accordingly, we examined whether MSAE could affect oxidative stress-mediated ROS generation, apoptosis, and Nrf2 activation in HaCaT keratinocyte cells.

## 2. Materials and Methods

### 2.1. Plant Materials and Extraction

*Mentha suaveolens* was collected from Jeju Island, South Korea. The dried *M. suaveolens* leaf sample was extracted with distilled water at 80°C. Then, *M. suaveolens* aqueous extract (MSAE) was filtered through a 5-*µ*m charcoal filter, frozen at −20°C, and lyophilized. The 200 g of dried *M. suaveolens* leaf sample produced 22 g of dried extract residue (11%). MSAE was dissolved in deionized water for further analysis.

### 2.2. Cell Culture and Treatment

HaCaT cells were maintained in Dulbecco's Modified Eagle's Medium (DMEM) supplemented with 10% fetal bovine serum (FBS) containing 1% penicillin-streptomycin and incubated at 37°C in humidified atmosphere with 5% CO_2_. All the cells were then cultured with DMEM containing 1% FBS for 18 h before harvest. Throughout this time, the cells were treated with various concentrations (10, 50, and 100 *μ*g/mL) of MASE. The control cells were incubated with water only.

### 2.3. Cell Viability Assay

The effect of MASE on the viability of HaCaT cells was measured by the MTT assay. Briefly, the cells were plated in a 96-well cell culture plate at a density of 1.8 × 10^4^ cells/well and allowed to attach for 24 h. After the cells were attached to the plate, different concentrations of MASE were used to treat the cells for 18 h followed by treatment with 1.6 mM H_2_O_2_ for 6 h. The cells were incubated with MTT reagent (1 mg/mL in PBS) for 2 h. Then, MTT solution was removed from the wells by aspiration, and formazan crystals were dissolved in 150 *μ*L of DMSO. The absorbance was measured at the wavelength of 570 nm using an ELISA microplate reader (Bio-Tek Instruments, Winooski, VT, USA).

### 2.4. Determination of Intracellular Reactive Oxygen Species

Intracellular ROS formation was detected using 2′,7′-dihydrofluorescein diacetate (DCFH-DA, Sigma chemicals Co., St. Louis, MO, USA). A nonfluorogenic compound DCFH-DA is oxidized to fluorescent compounds DCF by hydroxyl radical, peroxyl radical, and other reactive oxygen species (ROS) within the cell [15]. HaCaT cells were seeded at the density of 2 × 10^4^ in a 96-well black plate (Greiner Bio-one GmbH, Frickenhausen, Germany) for microplate reader and a chamber slide (ThermoFisher, Waltham, MA, USA) for fluorescence microscope.

After MSAE treatment for 18 h and 1.6 mM H_2_O_2_ treatment for 30 min, the cells were incubated with 25 *μ*M DCF-DA in culture medium at 37°C for 30 min in the dark. Intracellular ROS as indicated by DCF fluorescence was measured using an EVOS fluorescence microscope (ThermoFisher, Waltham, MA, USA) and an Infinite M200 microplate reader (Tecan, Männedorf, Switzerland) with excitation and emission wavelengths of 485 nm and 530 nm, respectively.

### 2.5. Western Blotting

The cells were lysed with Cytobuster protein extraction reagent (Novagen, Madison, WI, USA) (pH 7.4) containing protease inhibitor cocktail. Equal amounts of protein fractions (20 *μ*g) were loaded into each lane, separated by SDS-PAGE on 4∼12% Bis-Tris protein gels (ThermoFisher, Waltham, MA, USA), and then transferred onto polyvinylidene difluoride (PVDF) membranes. These membranes were blocked with 5% nonfat dried milk in Tris-Buffered Saline with Tween 20 (TBST) buffer for 1 h at room temperature and then incubation with primary antibodies overnight at 4°C. After incubating with a primary antibody, the membranes were incubated with a horseradish peroxidase- (HRP-) conjugated secondary antibody and visualized using an enhanced chemiluminescence (ECL) solution (ThermoFisher, Waltham, MA, USA). Western blot analyses with antibodies against *β*-actin, cleaved PARP, cleaved caspase-3, cleaved caspase-9, and HO-1 (Cell signaling, Danvers, MA, USA) were performed as described previously [16].

### 2.6. Antioxidant Response Element Reporter Assay

HaCaT cells were stably integrated with pGL4.37 Vector (Promega, Madison, WI, USA) using X-treme GENETM HP DNA transfection reagent (Roche Diagnostics, Risch-Rotkreuz, Switzerland). After selection using 0.2 *μ*g/mL hygromycin, one colony was cultured into a 24-well plate and subcultured sequentially into a 6-well plate for five weeks for hygromycin selection. The transfected cells were seeded at density of 6.5 × 10^4^ cells/well in a 6-well plate and treated with MSAE for 18 h followed by treatment with 1.6 mM H_2_O_2_ for 6 h. Luciferase activities were measured using a Dual Luciferase Reporter Assay System (Promega, Madison, WI, USA).

### 2.7. Quantitative Real-Time PCR

Total RNA was extracted using an RNeasy Mini Kit (Qiagen, Hilden, Germany) according to manufacturer's instruction, and cDNA was generated with cDNA Synthesis Platinum Master Mix (GenDEPOT, Barker, TX, USA). Relative gene expression analysis was performed using ABI PRISM 7300 (Applied Biosystems, Foster City, CA, USA). Briefly, 20 *μ*L of reaction mixture including 300 nM of each primer and 10 *μ*M of SYBR green PCR master mix (Applied Biosystems, Foster city, CA, USA) was subjected to 40 cycles of RT-PCR (95°C for 15 s, 55°C for 40 s, and 72°C for 10 min).

### 2.8. Preparation of Cytosolic and Nuclear Extracts

To evaluate Nrf2 translocation, the cells were used to obtain cytosolic and nuclear extracts using NEPER Nuclear and Cytoplasmic Extraction Reagents Kit (Thermo Scientific, Waltham, MA, USA). After incubating with primary antibodies against Nrf2, Lamin B1 (Abcam, Cambridge, UK), and *β*-tubulin (Sigma-Aldrich, St. Louis, MO, USA), the membranes were incubated with a horseradish peroxidase-conjugated secondary antibody (Cell signaling, Danvers, MA, USA) and visualized using an ECL solution (ThermoFisher, Waltham, MA, USA).

### 2.9. Statistical Analysis

Data are presented as means ± standard deviations (SD). Statistical significance was determined by a two-tail unpaired *t*-test using GraphPad PRISM. The critical level for significance was set at *P* < 0.05.

## 3. Results and Discussion

### 3.1. MSAE Inhibits H_2_O_2_-Induced Cytotoxicity in HaCaT Cells

**Figure 1:**
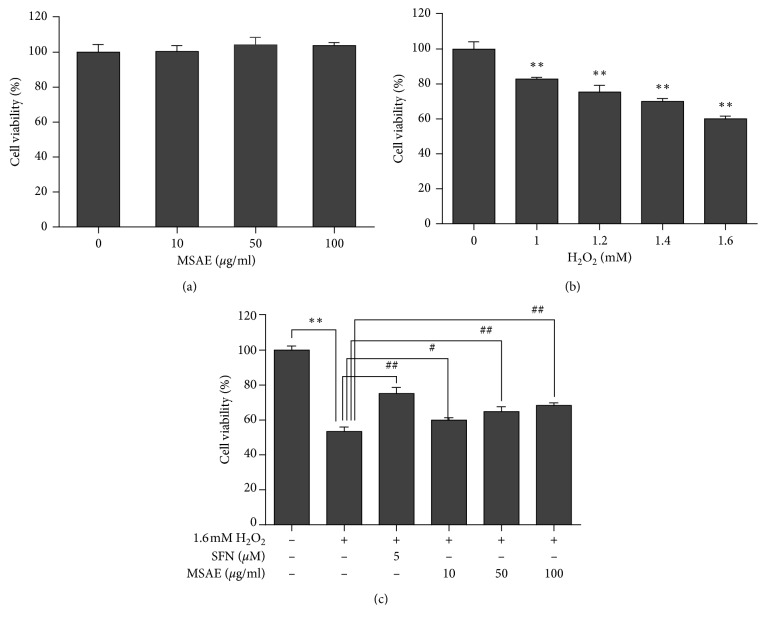
Effect of MSAE on cell viability in HaCaT keratinocyte cells. The cells were treated with different concentrations of MSAE for 18 h (a) or H_2_O_2_ for 6 h (b). (c) To measure the effect of MSAE on attenuation of H_2_O_2_-induced cell death, cells were pretreated with MSAE for 18 h followed by treatment with 1.6 mM H_2_O_2_ for 6 h. Values are expressed as mean±SD of three independent experiments. ^*∗∗*^, *p* < 0.01 compared with untreated cells; ^#^, *p* < 0.05; ^##^, *p* < 0.01 compared with H_2_O_2_-treated cells.

Cytotoxicities of MSAE and H_2_O_2_ to HaCaT cells were determined by the MTT assay. Treatment with MSAE for 18 h did not show cytotoxicity to HaCaT cells at concentration up to 100 *μ*g/mL (Figure 1(a)). However, H_2_O_2_ decreased HaCaT cell viability in a dose-dependent manner (Figure 1(b)). To explore the cytoprotective effect of MSAE, HaCaT cells were treated with MSAE for 18 h followed by treatment with 1.6 mM H_2_O_2_ for 6 h. Viability of HaCaT cells pretreated with MSAE followed by H_2_O_2_ treatment was higher than that of cells treated with H_2_O_2_ without such pretreatment (Figure 1(c)). Sulforaphane (SFN), a Nrf2 activator, served as a positive control [17–19].

### 3.2. Effect of MSAE on Intracellular ROS Generation in HaCaT Cells

**Figure 2:**
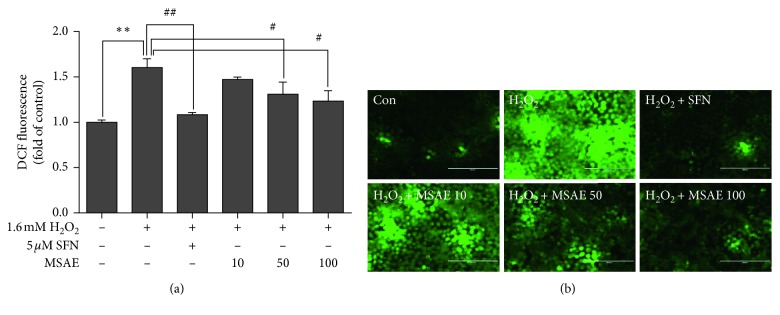
Effect of MSAE on ROS generation in HaCaT keratinocyte cells. Cells were pretreated with different concentrations of MSAE for 18 h or 5 *μ*M sulforaphane followed by treatment with 1.6 mM H_2_O_2_ for 30 min. These cells were incubated at 37°C in the dark for 30 min with culture medium containing 25 *μ*M DCF-DA. Intracellular ROS accumulation was detected by ELISA (a) and fluorescence microscopy. (b) Data are expressed as mean±SD of three independent experiments. ^*∗∗*^, *p* < 0.01 compared with untreated cells; ^#^, *p* < 0.05; ^##^, *p* < 0.01 compared with H_2_O_2_-treated cells.

To investigate mechanisms involved in the protective effect of MSAE, intracellular ROS generation was determined using DCF-DA. Fluorescence intensity and images of the cells were measured by using a microplate reader and fluorescence microscopy, respectively. These results showed that H_2_O_2_-treated HaCaT cells had increases of fluorescence, an indicator of intracellular ROS generation, compared with untreated cells. Treatment with MSAE prior to H_2_O_2_ exposure significantly reduced the production of intracellular ROS generation (Figures 2(a) and 2(b)).

### 3.3. MASE Modulates Apoptotic Gene Expression in H_2_O_2_-Treated HaCaT Cells

**Figure 3:**
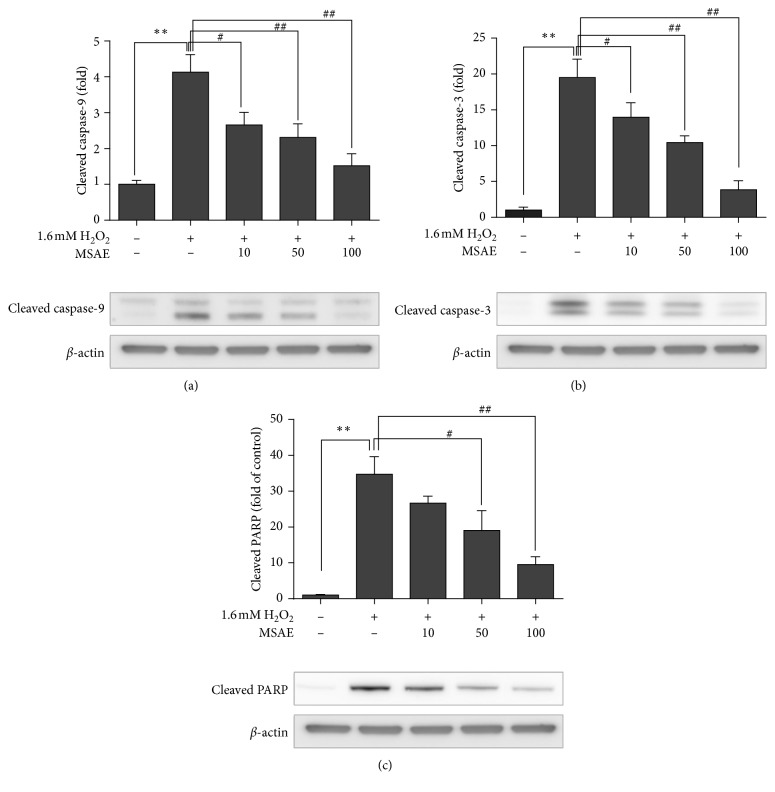
Effect of MSAE on apoptotic and antiapoptotic gene expression. (a) cleaved caspase-3, (b) cleaved caspase-9, and (c) cleaved PARP levels were measured to confirm MSAE's inhibition effects on H_2_O_2_-induced apoptosis. Data are presented as mean±SD of three independent experiments. ^*∗*^, *p* < 0.05; ^*∗∗*^, *p* < 0.01 compared with untreated cells; ^#^, *p* < 0.05; ^##^, *p* < 0.01 compared with H_2_O_2_-treated cells.

Caspases can trigger the apoptotic pathway. They are categorized into initiator caspases and effector caspases. Initiator caspases include caspase-2, caspase-8, caspase-9, and caspase-10 while effector caspases include caspase-3, caspase-6, and caspase-7 [20]. To further estimate whether the protective effect of MSAE on H_2_O_2_-induced cell death was due to inhibition of apoptosis, we analyzed the apoptotic-related gene expression. Treatment with 1.6 mM H_2_O_2_ induced the activation of proapoptotic proteins such as caspases. Levels of initiator caspase (caspase-9) and effector caspase (caspase-3) were decreased in the MSAE-treated group compared with those in the MSAE-untreated control group (Figures 3(a) and 3(b)). We also analyzed the inhibitory effect of MSAE on cleavage of PARP, a major substrate of both caspases [21]. Results showed that pretreatment with MSAE inhibited cleavage of PARP in a dose-dependent manner (Figure 3(c)).

### 3.4. MSAE Upregulates HO-1 Expression in HaCaT Cells

**Figure 4:**
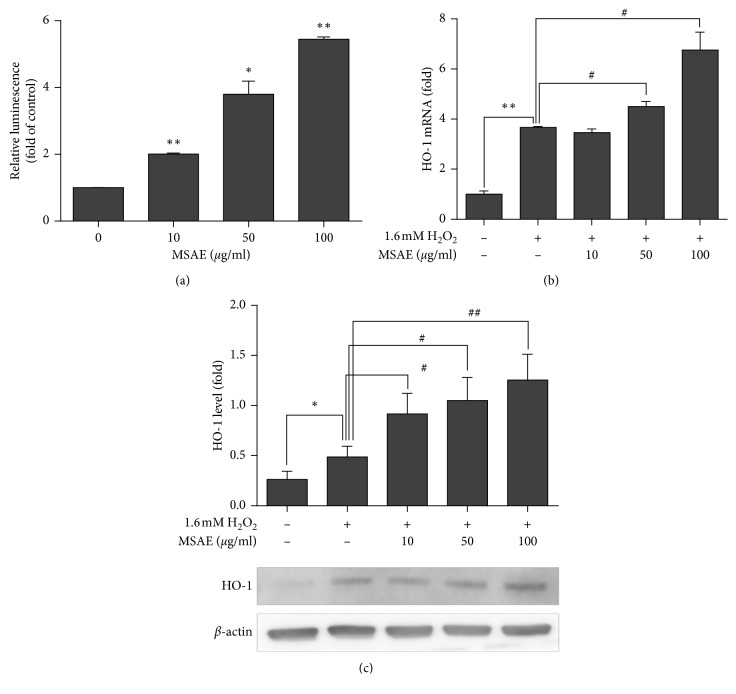
Induction of HO-1 expression by MSAE in HaCaT keratinocyte cells. After transfection with luciferase reporter plasmid, the cells were pretreated with different concentrations of MSAE for 18h. (a) ARE luciferase activity is presented as fold of untreated control±SD of three independent experiments. To analyze the effect of MSAE or H_2_O_2_ on HO-1 expression, HaCaT cells were treated with MSAE for 18 h followed by treatment with 1.6 mM H_2_O_2_ for 6 h. (b) Transcriptional levels were detected by RT-PCR. (c) Translational levels were detected by western blot analysis. Data are presented as mean ± SD of three independent experiments. ^*∗*^, *p* < 0.05 compared with untreated cells; ^#^, *p* < 0.05; ^##^, *p* < 0.01 compared with H_2_O_2_-treated cells.

To determine whether protective effects of MSAE against oxidative stress and apoptosis were due to the antioxidant gene induced by H_2_O_2_-mediated oxidative stress, the activity of ARE promoter was examined using luciferase reporter assay. Luciferase activity derived from the ARE promoter was increased in the group pretreated with MSAE (Figure 4(a)).

RT-PCR and western blot analysis were performed to examine the expression of phase II detoxifying enzymes regulated by Nrf2/ARE pathway such as HO-1 in HaCaT cells. Results of RT-PCR demonstrated that H_2_O_2_-meditated oxidative stress induced mRNA expression of HO-1. Interestingly, mRNA expression levels of HO-1 (Figure 4(b)) were increased by pretreatment with MSAE for 6 h. Similar to the results of mRNA expression levels, western blot results showed that protein levels of HO-1 were also increased by pretreatment with MSAE (Figure 4(c)).

### 3.5. MSAE Stimulates Nrf2 Nuclear Translocation in Oxidative Stress-Induced HaCaT Cells

**Figure 5:**
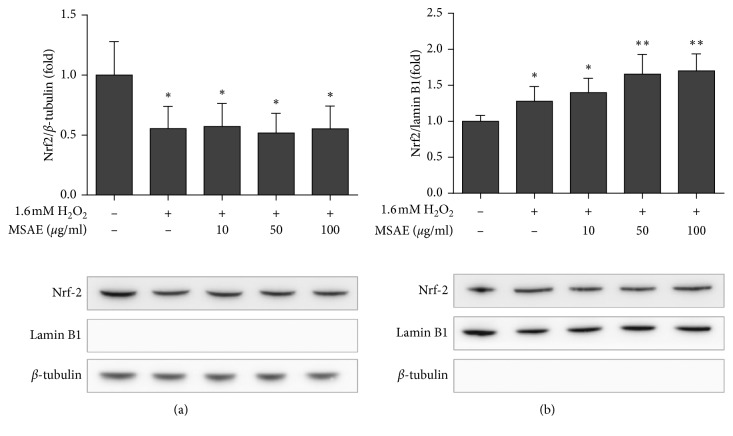
*Mentha suavelones* aqueous extract (MSAE) induces Nrf-2 translocation. The cells were pretreated with MSAE for 18 h followed by treatment with 1.6 mM H_2_O_2_ for 6 h protein levels of Nrf2 in the cytosol (a) and nucleus (b) were analyzed by western blotting. Data are presented as mean±SD of three independent experiments. ^*∗*^, *p* < 0.05; ^*∗∗*^, *p* < 0.01 compared with untreated cells.

Nrf2 plays an important role in the protection of skin cells. It binds to the ARE sequence in oxidative stress [22]. To determine the MSAE-mediated accumulation of Nrf2 in nuclear, Nrf2 proteins located in the cytoplasm and nuclear fraction were detected using western blot analysis. Nrf2 protein level present in the nuclear fraction was increased in cells treated with 1.6 mM H_2_O_2_ for 6 h after pretreatment with various concentrations of MSAE (10, 50, and 100 *μ*g/mL) for 18 h (Figure 5(b)). By contrast, Nrf-2 protein level in the cytoplasm fraction was decreased (Figure 5(a)). To confirm that there was no cross contamination in each fraction, the expression level of *β*-tubulin, a cytoplasmic marker, was monitored in the nuclear fraction. The expression level of nuclear protein Lamin B1 was determined in the cytoplasmic fraction. As shown in results, no cross contamination was detected.

## 4. Discussion

Oxidative stress is defined as an imbalance condition between the generation of ROS and antioxidant defenses. It is induced by the overproduction or inadequate removal of ROS. Skin is a major target of oxidative stress as ROS occurs after skin has a direct contact with the external environment. ROS play central roles in the incidence of skin disorders such as inflammation, photoaging (premature skin aging), immunosuppression, carcinogenesis, and apoptosis [1, 23]. In the present study, we examined the antioxidant capacity of MASE to prevent oxidative stress-induced cell damage and investigated the role of Nrf2/HO-1 pathway in protective effects of MSAE against H_2_O_2_-induced apoptosis in HaCaT cells. Our data from MTT assay showed that MSAE significantly rescued cell viability (Figures 1(a)–1(c)). Furthermore, under the condition of nontoxicity concentration, we found that elevated ROS accumulation in H_2_O_2_-exposed cells was substantially inhibited by MSAE pretreatment (Figures 2(a) and 2(b)), indicating free radical scavenging activity and protective properties of MSAE.

Elevated levels of ROS are associated with main pathways of apoptosis mediated by mitochondria. They are responsible for later mitochondrial events, leading to full activation of the caspase cascade [24]. According to their position in apoptotic signaling, caspases can be classified as initiator and effector caspases. The initiator caspase, also known as upstream caspase, includes caspase-2, caspase-8, caspase-9, and caspase-10. It is activated by its interaction with caspases adaptor. The effector caspase (downstream caspase) includes caspase-3, caspase-6, and caspase-7.

Cytochrome C is released from the mitochondrial inner membrane into cytosol. It forms a complex with apoptosis-activating factor-1 (Apaf-1) called an apoptosome which activates procaspase-9. Activated caspase-9 is able to cleave caspase-3 and -7 directly [25]. Cleaved caspase-3 plays a primary role in the cleavage of poly (ADP-ribose) polymerase (PARP), a nuclear protein that repairs damaged DNA during apoptotic cell death [18, 26]. In the present study, we assessed the antiapoptotic effect of MSAE via inhibition of apoptosis-related proteins such as cleaved caspase-9 (Figure 3(a)), cleaved caspase-3 (Figure 3(b)), and cleaved PARP (Figure 3(c)). These results showed that pretreatment with MSAE might prevent DNA damage induced by H_2_O_2_ and suppress mitochondrial caspase-related apoptosis pathway.

Skin has a variety of antioxidant systems to protect from harmful effects of oxidative agents. Protection of the skin against oxidative stress is performed by some enzymes which detoxify the free radicals. Superoxide dismutase (SOD), catalase (CAT), NQO-1, and HO-1 are the key enzymatic antioxidants by which the free radicals are removed against oxidative stress [27]. Expression levels of enzymatic antioxidants are induced by exposure to mild oxidative stress [28]. The moderate oxidative stress activates Nrf2 that then induces the transcription of enzymatic antioxidants containing ARE sequence in their promoter [22, 29]. The regulation of enzymatic antioxidants by Nrf2/ARE pathway might be a defense mechanism used to reduce ROS against oxidative stress. In our study, we observed that MSAE increase the ARE activity (Figure 4(a)) and tested whether MSAE increase the expression of SOD, CAT, NQO-1, and HO-1. Other expressions except of HO-1 was not affected by treatment with MSAE (data not shown). Therefore, we focused on the cytoprotective mechanism of MSAE against H_2_O_2_^−^induced oxidative stress by assessing the expression of HO-1. It is well known to be a major phase II detoxification enzyme which catalyzes the metabolism of heme into carbon monoxide, biliverdin, and ferritin. These by-products of heme are strong antioxidants. Through this process of breaking down heme, HO-1 can protect cells by net reduction of ROS [6, 30]. Similar to the results of previous studies, results of the present study showed that HO-1 expression in the H_2_O_2_-exposed group was increased than that in the control group. Furthermore, pretreatment with MSAE followed by H_2_O_2_ exposure upregulated HO-1 expression in HaCaT cells at both transcriptional and translational levels (Figures 4(b) and 4(c)).

The expression of HO-1 at the transcriptional level is regulated by ARE sequences located in the promoter region of Hmox 1. These sequences are binding sites for Nrf2, the major regulator of phase II detoxification enzymes. As a redox-sensitive transcription factor, Nrf2 is increased by overproduction of ROS (mainly hydrogen peroxide). It plays a pivotal role in cellular antioxidant defense by mediating the induction of a variety of antioxidant defense enzymes [22]. It has long been known that the mechanism of Nrf2/ARE activation involves the dissociation of the Keap1-Nrf2 complex in the cytoplasm [4, 31]. Keap1, a negative regulator of the Nrf2/ARE pathway, suppresses Nrf2-dependent transcription by forming a Keap1/Nrf2 complex in the cytoplasm under basal condition. However, under condition of oxidative stress, Nrf2 is separated from the Keap1-Nrf2 complex. It then translocates into the nucleus where it forms a complex with a small Maf protein and then binds to ARE sequences [32, 33]. In the present study, we investigated whether the protective effect of MSAE against H_2_O_2_-induced oxidative stress was attributed to Nrf2/ARE pathway in HaCaT cells. As shown in Figure 4(a), pretreatment with MSAE increased the ARE promoter activity in a dose-dependent manner. In addition, western blot assay demonstrated the effect of MSAE on translocation of Nrf2. As shown in Figure 5, pretreatment with MSAE accumulated nuclear Nrf2 levels. Therefore, MSAE may lead to Nrf2 translocation into the nucleus where it can activate the ARE promoter activity and transcription of antioxidant genes.

## 5. Conclusions

Our results demonstrate that MSAE can protect H_2_O_2_-treated human keratinocyte HaCaT cells against oxidative stress by decreasing intracellular ROS generation and apoptosis-related gene expression. This effect is also associated with the translocation of Nrf2 and upregulation of the expression of antioxidant gene HO-1. Findings of this study suggest that MSAE can reduce oxidative stress as a strong antioxidant candidate and alleviate various skin diseases associated with oxidative stress.

## Data Availability

The data used to support the findings of this study are available from the corresponding author upon request.
